# Think it through first: questions to consider in writing a successful grant application

**DOI:** 10.1007/s00247-014-3053-6

**Published:** 2014-11-19

**Authors:** Owen J. Arthurs

**Affiliations:** Department of Radiology, Great Ormond Street Hospital for Children NHS Foundation Trust, Great Ormond Street, London, UK WC1N 3JH

**Keywords:** Reseach funding, Paediatric radiology, Grant writing, National Institute for Health Research

## Abstract

Writing a good grant application is a skill that can be rehearsed in the same way as writing a research paper or performing a scientific presentation to a lay audience. An overview of grant writing is provided here, with particular focus on the consideration and preparation required for each step.

## Outline and approach

The ability to present a project’s unique selling points succinctly, comprehensively and accurately is crucial to its success, but this can take a lot of practice. Allow plenty of time for the process of careful planning, ideas, hypothesis evaluation, discussion with colleagues, and several rounds of improvement and evolution to tailor the grant to the point of submission.

Before starting to write, consider the following key questions:

1. Why is my project important and how is it different from others?
2. What resources do I need?
3. What type of grant do I need?
4. Who can provide this type of funding?
5. Why should they fund me?

Don’t invent a project to fit a funding stream (“chasing the money”), but instead try to use your scientific and clinical knowledge to outline your project and proposal, and then find money to fund your vision rather than the other way around. When grant writing is difficult, try to verbalise your ideas and then write down your exact words, using this as a starting point for your writing.

After a project idea has been formulated, the required resources often determine the type of grant that is required. Several types of grants are available, including small project grants (for equipment, imaging costs), personal fellowships (for salary costs, sometimes including project costs), project grants (for a combination of salary and project costs) and programme grants (for comprehensive project costs and salary for several staff members). Check which funding body offers to fund which type of project, because most grant streams are quite specific about what they will and will not fund, both in terms of subject matter as well as resources (equipment or staff costs). Before starting, check directly with a funding organisation as to whether your proposal is suitable. If necessary, they may be able to point you towards a more appropriate funding stream.

If you have never applied for a grant, it may be best to start small. For instance several local groups, smaller charities, UK Royal Colleges, etc., offer start-up funding (which is also called small project grants, seed funding, or junior grants). There may be small grants available specific to your career grade, such as start-up grants for clinical lecturers or young academics within 5 or 10 years of completing their doctorate. These form a useful platform from which to gain pilot data, which can then be synthesised into a larger grant application. Getting a small grant is in itself an accomplishment that indicates potential, and is viewed favourably when applying for further funding. Read some general advice about grant writing [1–5] before finding out what might be available [6–12]. Consider the experiences of someone who already holds that particular type of grant [13] and whether there is local advice available from your own trust or support network [14, 15].

## The 3 Ps

Grants are widely believed to focus around the three Ps — person, project and place. *The person* leading the project needs to have a strong curriculum vitae (CV) or background and clear potential to succeed, and to be applying appropriately for their career stage and aspirations. *The project* should be novel, with high scientific merit and with clear aims and objectives as well as a realistic timeframe. It should address a fundamentally important research question (or takes steps towards this). *The place* should be an institution that has a strong track record in research, with the appropriate expertise, recognised supervisors/trainers and facilities. Many projects include an element of training, whether specific methodological aspects or generic research skills, and so it should be clear what new skills you will learn and that the senior members of your team are in a position to provide this. The project and training environment should be able to provide a valuable research experience.

## Key components (the 5 Ws and the H)

Funding bodies are looking to fund good-quality research, from researchers who will deliver on time, which represents good value for money (not necessarily cheap), and will have a wide scope outside the immediate research community. This is the what, where, when (why now?), how, who (why me?) and why approach. Whilst a funding body may require that your grant request be presented in a particular format, most grant applications adhere to the outline that follows (Table 1).

**Table 1:** Sample grant outline (modelled on NIHR RfPB outline)

Section	What is required	Things to think about
Research details	Generic details of host organisation, etc.	
Research title	Descriptive yet concise State if pilot/feasibility study	Keywords, and how to phrase the title It should be clearly within the funding scope
Lead applicant details	Your details	Who is the most appropriate person to lead the project?
Lead applicant CV	2-page summary CV	This should make it clear that you are the appropriate applicant
Co-applicants	The appropriate senior, junior and multidisciplinary team	Consider who will be providing a service for the project, and who will be directly involved in the data. Grants are stronger when statisticians, health economists, etc., are named as co-applicants
Research & development office	Who is hosting the project?	Usually the NHS organisation or affiliated university
History of the application	Have you submitted this to the funding stream before?	How many attempts are allowed?
Case for support point 1	Aims and objectives Scientific summary Lay summary Background Why is this research needed now?	What is your research question and null hypothesis? Outline the problem, research question, methodology and expected impact. Summarise without jargon or difficult words The background should be punchy, with reasons why the research is necessary, highlighting the difficulties encountered by yourself or others
Case for support point 2	Research plan and methodology	Outline the entire study, including design, setting, sample size, intervention, data collection, analysis and outcomes. Think about feasibility, bias, and how much work is involved. How to tackle potential problems?
Case for support point 3	Dissemination, outputs and expertise	How are you going to publicise (not just publish) your data? Think about Web sites and other social media, patient support groups, targeted conferences and workshops
Specific requirements	e.g., patient and public involvement	These are specific to an individual funding body’s particular grant scheme, so find out as much as possible before you start
Management and governance	Who will oversee the project to make sure it runs ethically, and on time?	What might the project milestones be? What are the key ethical issues in the project?
Intellectual property	Is there a new IP expected, or might your findings conflict with pre-existing IP?	IP is expensive to investigate, and so you should think about pre-existing IP or asking for funding to copyright new IP. Think about who would own the IP
Finances	Budget your project	How many of what equipment or staff do I need, over what time period? Value for money is more important than absolute figures – but each grant has a maximum limit
Suggested reviewers	Who might be experts in this field who would give good objective feedback?	You should approach these people in advance to ask them
Supporting documentation / appendix	You may need to include images, diagrams, etc., that do not fit elsewhere in the application	

Adapted with permission from [1]

*CV* curriculum vitae, *IP* intellectual property, *NHS* National Health Service, *NIHR* National Institute for Health Research, *RfPB* Research for Patient Benefit

### Summary: a succinct outline of your proposal

This is akin to the “elevator pitch” to outline your unique selling point. Imagine you and your hospital director or National Health Service chief executive stepped into the lift on the ground floor together and you had 90 s to get your point across. This type of “selling yourself” may not come naturally and may need several rounds of practice. Verbalising your proposal helps to iron out inconsistencies. You will also need a lay summary version of your proposal for a non-scientific audience.

### The case for need: contextualise and quantify the problem

What is the question that I am addressing? Why is this project needed? What previous literature is available? How important, or how big, is the problem? What is being done by other groups? What type of study would be required in an ideal world to address this issue (the “best design” approach)? If your proposal were successfully funded and successful in answering its objectives, what would that mean in a wider context? What is needed to bring this project to a wider audience?

### The aim of this project, in context

What is the expected outcome, and what is the expected impact? Individual projects rarely stand alone and should be portrayed within the wider context of your institution, career intensions, and scientific community. Weaker grants are more likely to be funded if the institution or researcher is particularly strong, and strong scientific applications may fail when there is perceived to be limited potential.

### Methodology

The methodology section gives a skeleton or framework of the task and the resources required. How will potential sources of scientific bias be dealt with? It should also include rigorous statistical analysis and an estimation of the power of the study — e.g., how many participants will be needed to reach a conclusion with x% confidence? This may be the first time that you have thought about involving a statistician, but doing so could help with much more than a sample size, including study objectives and design, analyses and outcome. It is often at this stage that other relevant team members become necessary, including clinical trialists, epidemiologists, health economists and qualitative researchers.

### Pilot data

It is difficult to justify funding a theoretical problem but much easier to convince someone when you have tested your theories and demonstrated “proof of principle”. Whereas a feasibility study answers the question “can this study be done?” a pilot study is a miniature version of the whole study to test whether all the components can all work together (recruitment, test, follow-up, etc.), and it is designed to iron out the logistical issues.

### Budget

Financing your project appropriately is essential. You need to consider the people who will be needed, as well as materials, equipment, space and time. Try to justify your inclusion of everyone and everything, including who is accountable for what. Funding bodies look for a mix of personnel with the right expertise and skills and a strong track record. Under-estimating or exaggerating the team skills or cost of your project is inappropriate; you must ensure that everything you need to make the project succeed is either included in the grant or is clearly funded from elsewhere. If there are potential intellectual property issues, either new or conflicting, this needs to be carefully considered, and any necessary investigation needs to be included in the budget. Discussing your project early with your finance manager or equivalent is extremely useful.

## Generic guidance around grant writing

Find or talk to someone who has written a similar grant, particularly for this funding body.

Several funding bodies publish their remit or scope, and your project must fit neatly within their portfolio of studies. They may also publish lists of current successful grants, where you may be able to identify both helpful colleagues and potential conflicts of interest, and they may publish examples of successful grants (“sample grants”) to help you.

## Read the terms and conditions carefully

All grant applications come with several pages of assistance regarding how to complete the document. Funders take a dim view of those who stray outside their guidance, either by asking for inappropriate items or by not conforming to the desired funding stream (remit or scope). Most funders expect to be telephoned for advice regarding the application process, and whilst they will not give specific advice, they can steer you in a helpful direction. Explicit requirements (e.g., patient involvement) should be clearly outlined and not included as an afterthought.

## Keep the writing clear and the reader engaged

You should try to clearly and concisely outline the problem, in the context of the current scientific literature, and demonstrate a clear need for your research. The style of writing can influence reader engagement. Appropriate punctuation, use of headings, boldface or italics for emphasis, and bullet points can help keep the reader (and author) focussed. Avoid technical jargon and unusual acronyms, but do include buzzwords.

## If at first you don’t succeed, try again and again

Many funding bodies publish success rates, which may be as low as 5–10%. When a grant application is turned down, try to get feedback as to why it wasn’t successful. Did you anticipate the potential problems in the grant that the funding body identified? Did you show that you had recognised these and thought how they might be dealt with, if encountered? Some funding bodies provide limited feedback and allow a limited number of repeated attempts at each stage — it is worth knowing how many attempts you have in advance of your first one. Several larger grant applications are now a two-stage process: an initial outline stage, and then a full application. It is strongly advised that you have written the full application by the time the outline deadline has arrived.

## Success! The next steps

There are several requirements for getting a project up and running other than successful funding. These include a sponsor (the organisation accountable for the research), ethical approval from a recognised body (to protect research participants), local research and development approval or permission, local research networks, as well as other regulatory approvals (e.g., clinical trials, medicines) and honorary contracts for those involved. Applying for these may be just as time-consuming (if not more so) than the actual grant application process and should ideally be undertaken in parallel. Getting the grant is just the beginning of your research journey, and whilst you are enjoying the research, bear in mind that all grants are finite, with a clear end date, and you will need to justify the use of resources.

## Conclusion

Although the grant writing process may seem daunting, help is widely available at each step, from conception through to submission. Your local research and development team, fellow scientists (especially those who sit on grant review panels), funding bodies and several Web sites are useful sources of guidance regarding where to start, and they might offer to review your proposals and give helpful feedback. The earlier you start, the more detailed feedback you will get, and the more time you have to respond to that feedback, so the better your application will be.
